# Where, when, and how the diagnosis of human visceral leishmaniasis is defined: answers from the Brazilian control program

**DOI:** 10.1590/0074-02760190253

**Published:** 2019-10-31

**Authors:** João Gabriel Guimarães Luz, Amanda Gabriela de Carvalho, Danilo Bueno Naves, João Victor Leite Dias, Cor Jesus Fernandes Fontes

**Affiliations:** 1 Universidade Federal de Mato Grosso, Instituto de Ciências Exatas e Naturais, Curso de Medicina, Rondonópolis, MT, Brasil; 2 Universidade Federal de Mato Grosso, Faculdade de Medicina, Programa de Pós-Graduação em Ciências da Saúde, Cuiabá, MT, Brasil; 3 Universidade Federal dos Vales do Jequitinhonha e Mucuri, Faculdade de Medicina, Teófilo Otoni, MG, Brasil; 4 Faculdade de Ciências Biomédicas de Cacoal, Curso de Medicina, Cacoal, RO, Brasil

**Keywords:** visceral leishmaniasis, kala-azar, diagnosis, public health, primary health care, Brazil

## Abstract

**BACKGROUND:**

Timely diagnosis is recommended by the Brazilian Visceral Leishmaniasis (VL) Surveillance and Control Program to reduce case fatality. Attempts at assessing this topic in Brazil are scarce.

**OBJECTIVE:**

This study aimed to describe where, when, and how the diagnosis of VL has been performed in a Brazilian endemic setting.

**METHODS:**

Data of all autochthonous cases confirmed between 2011 and 2016 (N = 81) were recorded. The care-seeking itinerary until the confirmation of VL diagnosis was assessed among 57 patients.

**FINDINGS:**

The majority of VL cases (79.1%) were reported by referral hospitals. The patients mainly sought primary health care centres at the onset of symptoms. However, they had to visit seven health services on average to achieve a confirmed diagnosis. The time from the onset of symptoms to the diagnosis of VL (T_D_) ranged from 1-212 (median, 25) days. The T_D_ was longer among adult patients. There was a direct correlation between the patient’s age and T_D_ (*r* = 0.22; p = 0.047) and a higher occurrence of deaths due to the disease among older patients (p = 0.002). Almost all the patients (98.9%) underwent laboratory investigation, and the VL diagnosis was mainly confirmed based on clinical-laboratory criteria (92.6%). Positive results for the indirect fluorescence antibody test (22.7%) and parasitological examination plus rk39-based immunochromatographic tests (21.3%) were commonly employed.

**MAIN CONCLUSIONS:**

VL diagnosis was predominantly conducted in hospitals with a long T_D_ and wide application of serology. These findings may support measures focused on early diagnosis, including a greater involvement of the primary health care system.

Visceral leishmaniasis (VL), or kala-azar, is a neglected tropical disease caused by protozoa of the genus *Leishmania* that are transmitted to humans and other mammals via the bite of female phlebotomine sand flies.^1^ In humans, the disease is clinically characterised by prolonged fever, weight loss, weakness, hepatosplenomegaly, hypergammaglobulinemia, and pancytopenia. If not treated appropriately, VL can progress to profound cachexia, systemic inflammation, bacterial infection, bleeding, and death.^2^

Brazil is one of six countries that share 90% of the VL burden worldwide, in addition to being the main endemic area for the disease in the Americas.^1^ Since the 1980s, VL has alarmingly been spreading to Brazilian urban centres as a zoonotic disease caused by *Leishmania infantum*, transmitted by the phlebotomines *Lutzomyia longipalpis* and *Lutzomyia cruzi*, with dogs being the main reservoir host.^3^ ^,^ ^4^ Presently, autochthonous VL cases have already been notified in 25.0% of Brazilian municipalities in almost all the states,^5^ with an average annual incidence of 3,500 cases.^6^

Late diagnosis has been identified as an important factor usually associated with death due to VL.^7^ ^,^ ^8^ ^,^ ^9^ Therefore, along with vector control and reservoir management, the Brazilian Ministry of Health strongly recommends early case finding followed by opportune treatment through the Visceral Leishmaniasis Surveillance and Control Programme (VLSCP).^3^ Thus, the national public health care system (Sistema Único de Saúde ― SUS) is set up to guarantee this. Basically, primary health care centres - basic care units (BCUs) or emergency care units (ECUs) - are essential for timely VL suspicion and confirmation. In addition, they should trigger case management, and depending on the clinical picture, patients may be redirected to tertiary care centres, such as referral hospitals.^1^ ^,^ ^10^

Despite the existence of specific patient management and treatment routines, the fatality rate due to VL has been increasing significantly in the country.^6^ ^,^ ^10^ From 2007 to 2014, 1,779 deaths were recorded among VL cases.^6^ In addition, Martins-Melo et al.^11^ recently estimated the national case-fatality rate due to the disease at 8.1%.

Given that early recognition is important for a better prognosis,^12^ and that attempts to assess the VL diagnosis in Brazilian endemic areas are scarce,^13^ the present study aimed to address this issue in the municipality of Rondonópolis, an important setting that recently emerged as highly endemic for the disease in Brazil.^5^ ^,^ ^14^ By unveiling where, when, and how the diagnosis of VL has been performed, we may provide useful information for the planning of integrated public policies towards the improvement of patient management and reduction of the death rate due to VL.

## MATERIALS AND METHODS

*Study area and design* - This retrospective and descriptive study assessed the aspects of VL diagnosis in the municipality of Rondonópolis (16º28’15”S and 54º38’08”W), located in the southern part of the state of Mato Grosso in Central-Western Brazil. It occupies an area of 4,159.12 km² and has an estimated population of 228,857 inhabitants.^15^ The local public health services comprise two referral hospitals, two ECUs, and 37 BCUs.

According to the Brazilian Ministry of Health, Rondonópolis is classified as an area with intense transmission of VL.^16^ Between 2003 and 2016, 210 human cases were reported there.^17^ Although the control measures of the VLSCP have been conducted locally, the incidence and case fatality due to VL recently reached peaks of 12.1 cases/100,000 inhabitants and 20.0%, respectively.^14^ In addition, a recent publication detected a high seroprevalence of canine VL in the urban area of the municipality.^17^

*Data collection* - In Brazil, VL is a notifiable disease. Thus, when a clinical suspicion is made, the health service where the patient was attended to should immediately fill in a specific form of the Brazilian Notifiable Diseases Information System (Sistema de Informação de Agravos de Notificação). This form collects epidemiological and clinical data, and triggers the investigation of the case by the local surveillance department. During this process, additional information regarding diagnosis, treatment, and outcome are included on the form.^3^

In the present study, secondary data were collected by the individual analysis of all these VL notification/investigation forms obtained from Sistema de Informação de Agravos de Notificação, which is coordinated by the Epidemiological Surveillance Sector of the Municipal Health Department of Rondonópolis. The diagnostic aspects of all autochthonous VL cases reported and confirmed in the municipality among resident individuals between 2011 and 2016 were considered (N = 81), for whom epidemiological and clinical characteristics were already described.^14^ Relapses or cases reported in duplicate were excluded. The following information was recorded for each VL patient: date of birth, date of onset of symptoms, date of notification, source of notification, diagnostic confirmation criteria, laboratory methods employed, and outcome.

In addition, from March to October 2017, the patients were interviewed to assess the care-seeking itineraries until the VL diagnosis was confirmed. Briefly, they were asked about the number and types of health facilities visited until the diagnosis of VL was confirmed. The parents or legal guardians of individuals younger than 18 years at the time of data collection were interviewed on behalf of the patients.

*Data analysis* - Data were firstly tabulated and double-checked in spreadsheets using Microsoft^TM^ Excel 2013 (Microsoft Corp., Santa Rosa, CA, USA). Then, descriptive statistical analyses were performed for each variable, and data normality was assessed using the Shapiro-Wilk test. To compare simple proportions, their 95% confidence intervals (CIs) were estimated using the Wald method. In addition, the time between the onset of symptoms and the diagnosis of VL (T_D_) was determined for each patient. For this, the date of notification was considered as the date of VL diagnosis. The correlation between T_D_ and the patient’s age was evaluated using Spearman’s correlation coefficient and differences between the patients’ ages and the outcomes after treatment (dead due to VL or not) were analysed using the Mann-Whitney *U* test. Analysis items with p < 0.05 were considered statistically significant. Finally, the overall positivity rate for each diagnostic test was calculated by dividing the number of individuals with positive results in a given test by the number of individuals subjected to the given test expressed as a percentage. Data analysis and graphing were performed using STATA/SE 11.0 (StataCorp LP, College Station, TX, USA) and Prism 7.04 (GraphPad Software, La Jolla, CA, USA), respectively.

*Ethical considerations* - This study was approved by the Ethical Committee for Human Research of Júlio Müller University Hospital (CAAE number: 52023215.5.0000.5541).

## RESULTS

**Fig. 1:**
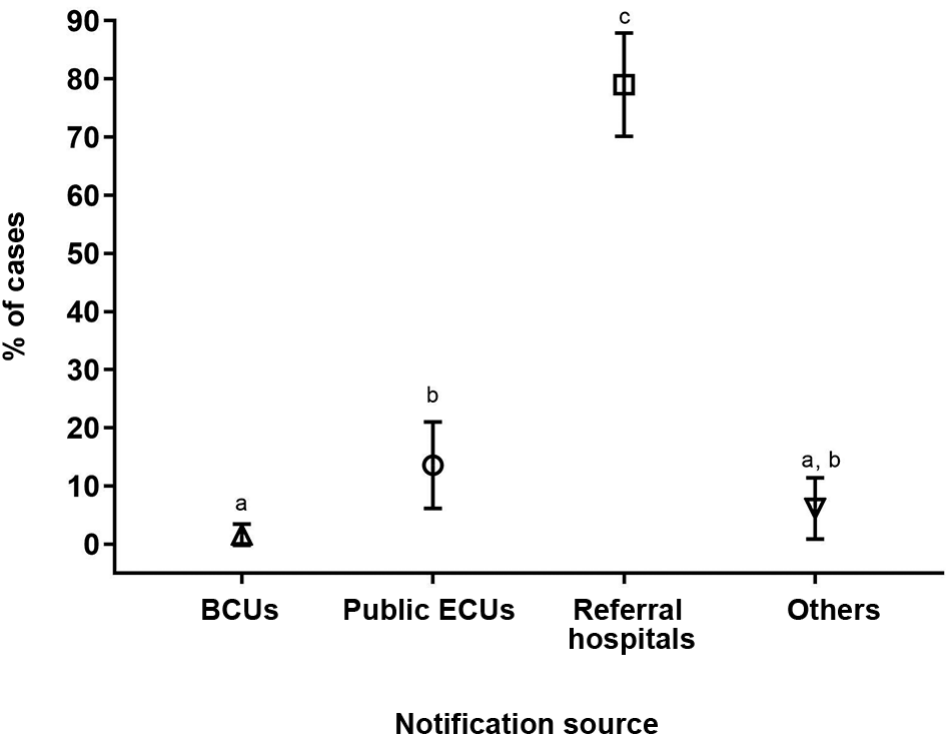
percentage distribution of the cases of visceral leishmaniasis according to the source of notification in the municipality of Rondonópolis, Mato Grosso state, Brazil (2011-2016). Vertical bars denote 95% confidence intervals of the proportions and the superscript letters indicate differences between proportions. BCUs: basic care units; ECUs: emergency care units.

In Rondonópolis, the majority of VL cases during the study period were significantly reported by referral hospitals (64/81, 79.1%; 95% CI, 70.1-87.9). Primary healthcare services, such as public ECUs and BCUs reported only 13.6% (12/81) (95% CI, 6.1-21.0) and 1.2% (1/81) (95% CI, 0.0-3.6) of cases, respectively (Fig. 1).

**Fig. 2:**
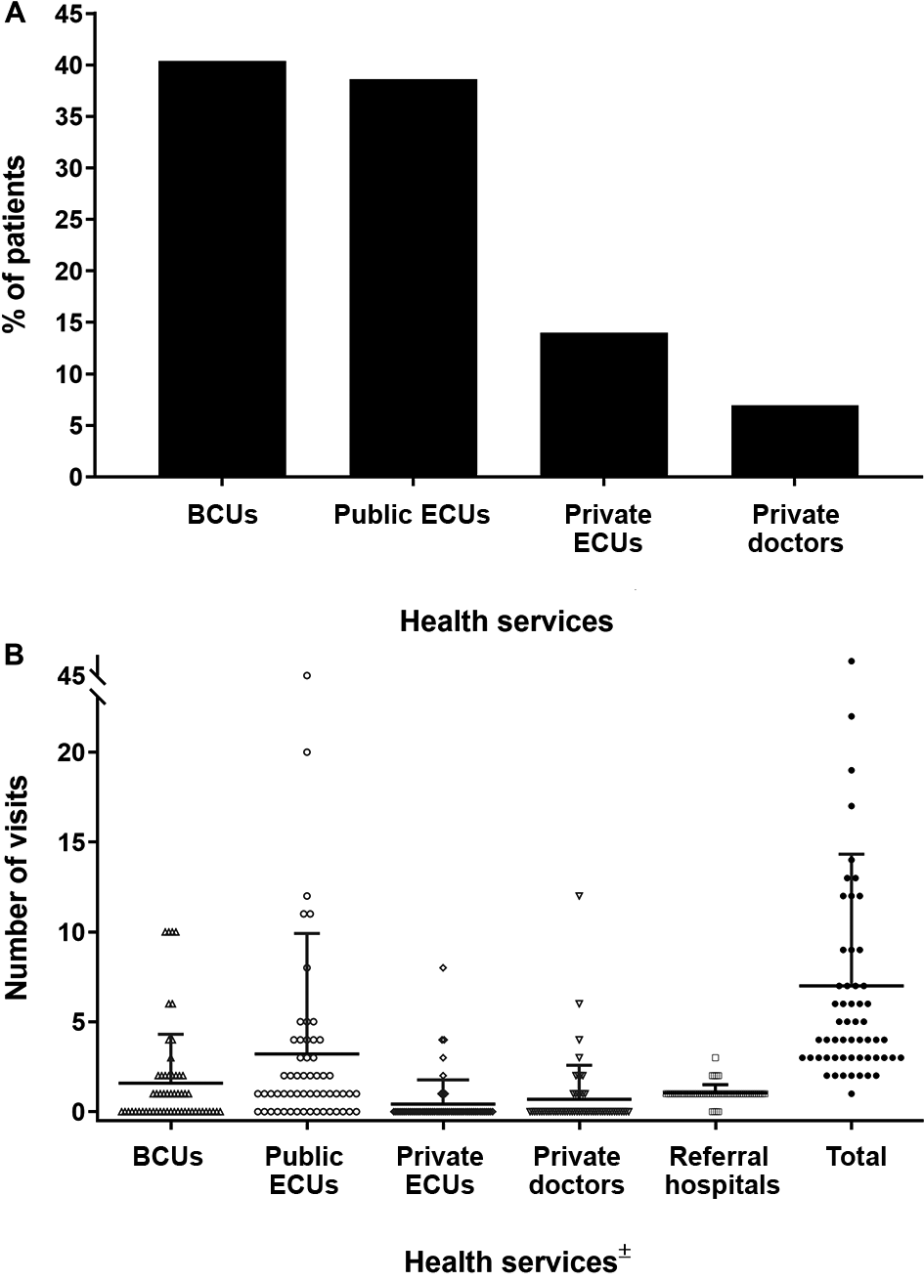
care-seeking itineraries until the confirmation of the diagnosis of visceral leishmaniasis (VL) in the municipality of Rondonópolis, Mato Grosso state, Brazil (2011-2016). This information was obtained from 57 patients. (A) represents the percentage distribution of the patients according to the first health service initially sought after the onset of VL symptoms. (B) shows the absolute (dots) and average (horizontal lines) number of visits to each health service until a confirmed diagnosis of VL was achieved. Vertical bars denote standard deviations. ±: the records of three patients who visited public doctors were not represented; BCUs: basic care units; ECUs: emergency care units.

Through interviews, it was possible to obtain information from 70.4% (57/81) of the patients about the number and types of health services visited until the diagnosis of VL. Of these, 40.4% (23/57) initially presented with clinical symptoms of VL at BCUs, followed by public ECUs (38.6%, 22/57), private ECUs (14.0%, 8/57), and private doctors (7.0%, 4/57) (Fig. 2A). Subsequently, these patients returned on average 3.2 [standard deviation (SD), 6.7] times to public ECUs, 1.6 (SD, 2.7) times to BCUs, 1.0 (SD, 0.4) time to a hospital, and 0.7 (SD, 1.9) times to private doctors before the diagnosis was confirmed. Therefore, the patients had to go to 7.0 (SD, 7.3) health facilities in average to confirm the diagnosis of VL (range, 1-46 health facilities) (Fig. 2B). A detailed characterisation of the individual care-seeking itineraries is described in Supplementary data.

**Fig. 3:**
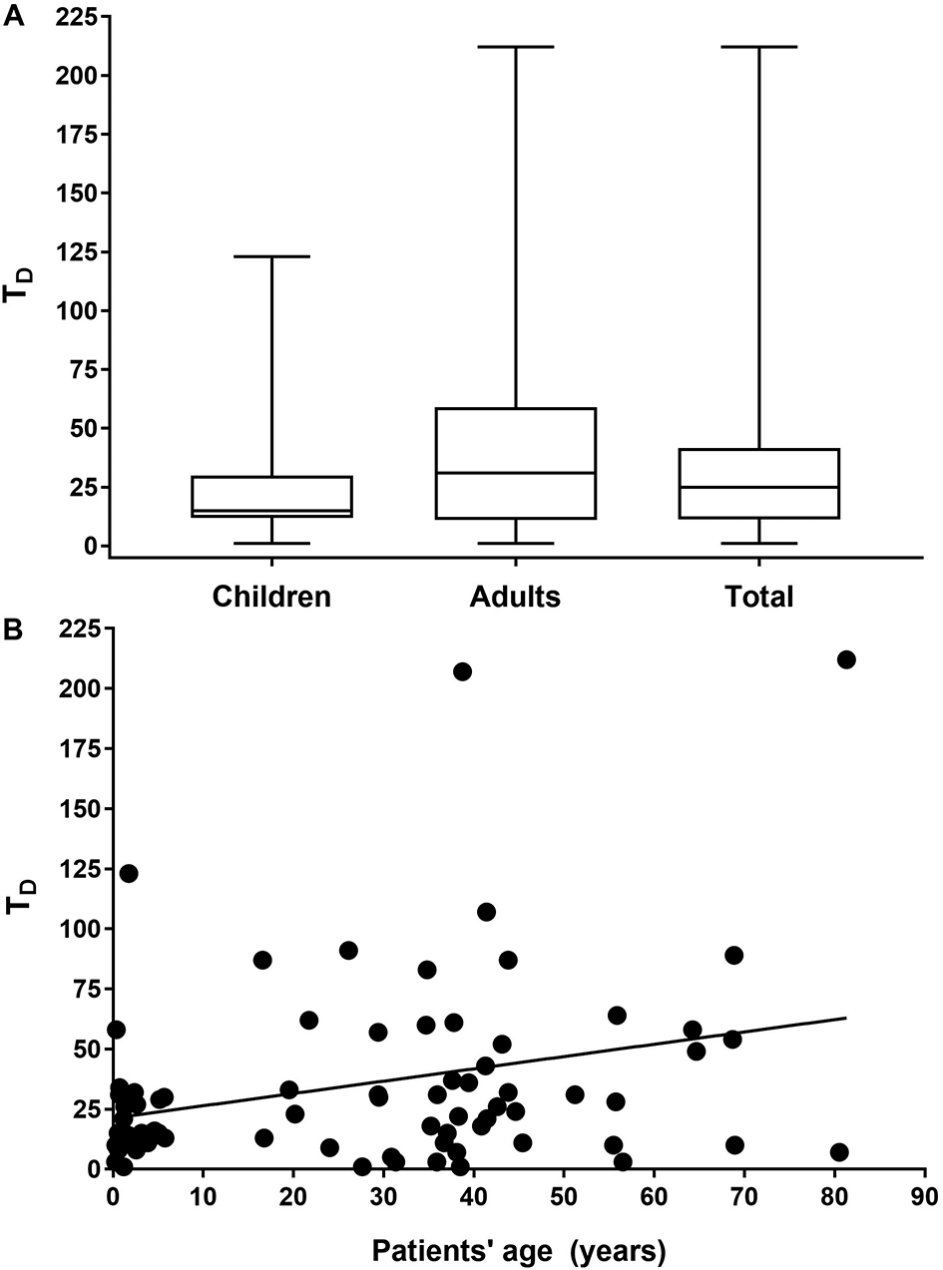
time between the onset of symptoms and the diagnosis of visceral leishmaniasis (VL) (T_D_) in the municipality of Rondonópolis, Mato Grosso state, Brazil (2011-2016). This information was obtained from 80 patients. (A) shows the box plot of T_D_ for children, adults and total (children + adults) patients. (B) represents the scatter diagram of the T_D_ according to patients’ ages.

The T_D_ ranged from 1-212 (median, 25) days. However, such parameters varied widely when children or adolescents (age, 0 - 18 years) (15 days) were compared to adult patients (age > 18 years) (31 days) (Fig. 3A). Indeed, there was a direct correlation between the patient’s age and T_D_ (*r* = 0.22; p = 0.047) (Fig. 3B). In addition, a higher age was observed among patients who died due to VL (median age, 55.9 years) when compared to the others (median age, 24.0 years) (p = 0.002) (data not shown).

**Fig. 4:**
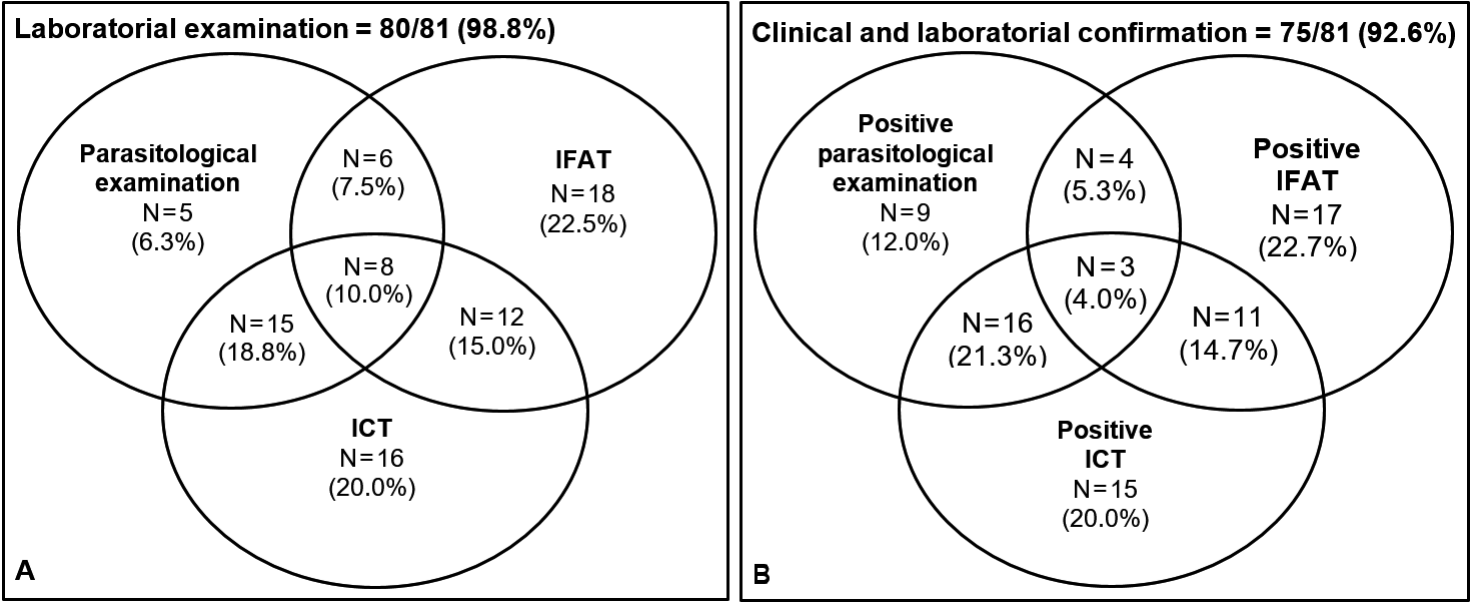
laboratory methods employed for the diagnosis of visceral leishmaniasis (VL) in the municipality of Rondonópolis, Mato Grosso state, Brazil (2011-2016). (A) represents the absolute and relative distribution of the 80 patients subjected to laboratory investigation for the diagnosis of VL according to the employed tests, either alone or in combination with two or three techniques at the same time (intersections). (B) represents the absolute and percentage distributions of the 75 patients whose diagnoses were confirmed using clinical and laboratory criteria according to the positivity in each of the employed tests, either alone or in combination with two or three techniques simultaneously (intersections). IFAT: indirect fluorescence antibody test; ICT: rk39-based immunochromatographic tests.

Almost all the patients (80/81, 98.8%) underwent an aetiological laboratory examination to investigate the diagnosis of VL. A total of 51, 44, and 34 individuals were tested using rk39-based immunochromatographic tests (ICT), indirect fluorescence antibody test (IFAT), and parasitological examination, respectively (Fig. 4A). These tests were performed alone or in combination with two or three techniques. In this way, IFAT alone (18/80, 22.5%) and ICT alone (16/80, 20.0%) were commonly used, followed by a combination of parasitological examination and ICT (15/80, 18.8%).

The diagnosis of VL was confirmed based on clinical and laboratory evidence in 92.6% (75/81) of cases, and based on clinical and epidemiological features among the remaining patients. Parasitological examination demonstrated the highest overall positivity rate (32/34, 94.1%), followed by ICT (45/51, 88.2%), and IFAT (35/44, 79.5%). Nonetheless, the most common laboratory techniques used for diagnostic confirmation were IFAT alone (17/75, 22.7%), a combination of parasitological examination and ICT (16/75, 21.3%), and ICT alone (15/75, 20.0%). Notably, only 4.0% (3/75) of the patients demonstrated concomitant positivity for the three techniques (Fig. 4B).

## DISCUSSION

While the efficacy of canine euthanasia and vector control are still controversial in controlling VL in Brazil,^5^ ^,^ ^13^ the early diagnosis and treatment of human cases seem to be an obvious measure for improving patient management and reducing the case fatality.^3^ However, the present study raised concerns that may be adversely affecting the goals of World Health Organization and the VLSCP.^1^ ^,^ ^3^ ^,^ ^10^

Given that the early clinical manifestations of VL tend to be mild, patients usually seek assistance at the front line of the SUS (BCUs and ECUs), which in turn should clinically suspect and report the disease.^1^ However, our study identified referral hospitals as the main sources of VL notification. According to Barbosa et al.,^18^ this may be explained by the inability of the health services network to establish effective principal portals of entry. However, the data obtained by interviewing VL patients strongly suggested that the local primary care services were unable to suspect the disease promptly.

Although the patients reported seeking care at public ECUs and BCUs at the onset of VL symptoms, the majority had to visit several additional health facilities before a clinical suspicion was raised and a correct diagnosis was made. The failure of primary health care services to promptly recognise VL may be attributed to the non-specific clinical symptoms presented by the patients, which usually overlap with those of other febrile syndromes.^19^ ^,^ ^20^ Moreover, there is still a lack of health professionals prepared to act on the recognition of VL and patient care.^18^ ^,^ ^21^ Lastly, the majority of available laboratory methods present limitations in both availability and feasibility in primary care establishments.^1^

It is expected that a patient seeking medical assistance several times will present a higher time lag between the beginning of clinical manifestations and the correct diagnosis of VL.^22^ In fact, similar to a multi-centric evaluation previously conducted in Brazil,^23^ the present investigation detected a high T_D_ in the study area. Although high, the T_D_ was probably underestimated, mainly because it requires information reported by the patient that is often imprecise. Thus, it should be considered that the onset of VL symptoms tends to be insidious, and that the affected population usually has a low educational level.^14^ ^,^ ^22^ Furthermore, we used the date of notification to determine T_D_, which was not necessarily equivalent to the period during which VL was confirmed.

This marked delay in diagnosis deserves attention because it may be associated with poor prognosis, worse treatment outcome, and/or death among VL patients.^8^ ^,^ ^9^ ^,^ ^24^ It is noticeable that signs of severe disease, such as jaundice, edema and bleeding, were recently reported with high frequency among VL cases from Rondonópolis.^14^ On the other hand, although the importance of humans in the transmission of *L. infantum* to sand flies in Brazil is still debatable, it can be speculated that this late diagnosis could contribute to VL transmission without the participation of animal reservoirs.^25^

Adult patients presented the highest median T_D_, which in turn was also directly correlated with the individual’s age. The differences among age groups and VL detection have been reported previously.^26^ The explanations for these results probably lie in aspects related to health personnel and/or patients. According to Caldas et al.,^26^ physicians tend to identify the disease faster and more easily among children, because they represent the age group known to be most affected by VL. In addition, parents usually seek healthcare for their children immediately after the onset of any clinical abnormality, particularly fever,^27^ which represents the most classical initial manifestation among VL patients.^14^ Delayed diagnosis among adult patients should be highlighted, since we also observed a higher occurrence of deaths due to VL among them.

The execution of laboratory tests is of paramount importance to help in the confirmation of VL diagnosis.^20^ Fortunately, almost all of the patients were subjected to etiological laboratory examination in Rondonópolis. It is likely that IFAT and ICT were frequently performed because of their non-invasive nature and easier execution when compared to parasitological examination. Another possible explanation is that the Brazilian Ministry of Health freely distributes ICTs to the states.^28^ However, during the study period, the use of such tests was limited to the referral hospitals in the study area. This may have contributed to delays in the diagnosis of VL.

As also observed by Spir et al.,^28^ clinical and epidemiological criteria were employed to define the diagnosis in very few patients. The association of a suggestive clinical picture and positive laboratory test results is particularly recommended to confirm VL.^3^ This is essential to avoid unnecessary administration of conventional treatment, which has significant toxicity and contraindications.^24^ In particular, serological positivity was widely employed as a criterion for VL confirmation in most cases.

However, both serological tests presented lower overall positivity rates than did the parasitological examination. This is in contrast to the well-known low sensitivity of direct identification of amastigotes in bone marrow, which is limited to 53-86%.^24^ The low overall positivity rate for ICT may be related to the high frequency of paediatric patients.^14^ Freire et al.^29^ recently observed a lower sensitivity of Kalazar Detect^TM^ among children, which was the ICT available in the scope of Brazilian public health for diagnosing VL during most of the study period.

This study has the following limitations: (i) secondary data are susceptible to a lack of information or uncontrollable errors in recording; (ii) it was not possible to collect information regarding the health services sought by all the patients; and (iii) such data may have been influenced by memory bias or overestimation of the information.

Despite these limitations, we believe that the worrying panorama regarding where, when, and how the diagnosis of VL has been performed may be extrapolated to other endemic areas. Although the studied population was limited to one single municipality, Rondonópolis has socioeconomic and demographic characteristics similar to other localities that have recently undergone urbanisation and expansion of VL in Brazil and abroad. Thus, our results may be useful to health authorities in the planning and execution of integrated public policies based on the improvement of early VL detection and patient management.

Incidentally, since 2015, the Brazilian Ministry of Health has incorporated rapid test kits for the diagnosis of VL into the *SUS*, which allowed immediate and accurate results to be obtained using whole blood samples.^30^ Nonetheless, it is essential to ensure the continuous supply of these test kits at primary health care centres. Continuous training of health professionals regarding leishmaniasis is also important, as the clinical diagnosis of VL is a difficult task that requires the involvement of the entire primary health care team.^1^ ^,^ ^21^ In this sense, the development and validation of diagnostic algorithms for a standardised clinical case definition of VL in BCUs and ECUs would be helpful.^20^

Targeted community engagement, such as health education programmes focused on disease recognition among the population at risk, is recommended.^1^ Moreover, a better articulation of the health service networks is important in the management of patients with clinical suspicion of VL.^21^ For this, it must be considered that the disease affects the poorest of the poor, and leads to progressive weakness among the individuals, which may compromise their ability to move unnecessarily between different health facilities. Finally, research focused on new diagnostic tools that fit the diverse contexts in which the disease occurs should also be encouraged, especially point-of-care tests, both serological and hopefully parasitological.

*In conclusion* - The diagnosis of VL occurred more frequently in referral hospitals in the study area, although the patients initially sought care at primary health care centres. This probably led to the long interval identified between the onset of symptoms and the detection of the disease, which was remarkable among adult patients. Clinical and laboratory criteria were predominantly employed for the confirmation of VL. For laboratory diagnosis, serology had greater importance.

These findings may support measures focused on the early recognition of VL, including a greater involvement of the primary health care system with continuous professional training, uninterrupted provision of point-of-care tests, and community engagement. Lastly, the development of more accurate diagnostic tools with application in diverse contexts should be encouraged.
