# Supplementary material for: Where, when, and how the diagnosis of human visceral leishmaniasis is defined: answers from the Brazilian control program
Source: Mem Inst Oswaldo Cruz. 2019 Oct 31;114:e190253. doi: 10.1590/0074-02760190253 (PMC6821129; doi:10.1590/0074-02760190253)
Supplement: Supplementary file 1 [file 1678-8060-mioc-114-e190253-s.pdf]

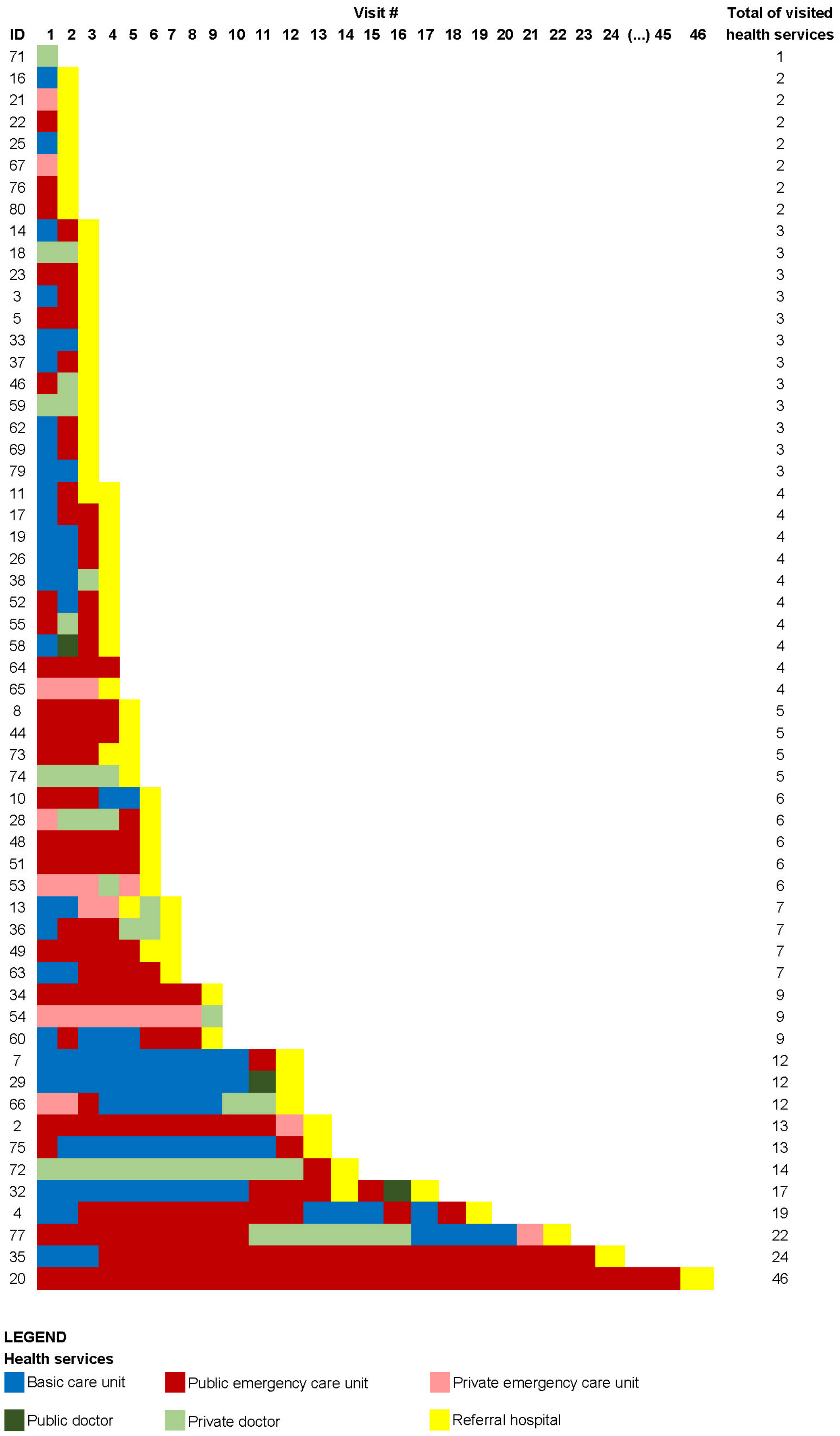

Individual care-seeking itineraries until the confirmation of the diagnosis of visceral leishmaniasis in the municipality of Rondonópolis, Mato Grosso state, Brazil (2011-2016).

cc BY
